# Health beliefs mediates the association between the number of non-communicable diseases and preventive behaviors in middle-aged and older adults in southern China

**DOI:** 10.1007/s40520-025-02939-3

**Published:** 2025-02-25

**Authors:** Yali Huang, Tingjun Wang, Huajun Wang, Yongjun Zeng, Liangdi Xie

**Affiliations:** 1 Fujian Hypertension Research Institute, The First Affiliated Hospital of Fujian Medical University, Fuzhou, 350005 China; 2 The School of Public Health, Fujian Medical University, Fuzhou, China; 3 Department of General Practice, National Regional Medical Center, Binhai Campus of the First Affiliated Hospital, Fujian Medical University, Fuzhou, China; 4 Department of General Practice, The First Affiliated Hospital of Fujian Medical University, Fuzhou, China; 5 Department of Geriatrics, The First Affiliated Hospital of Fujian Medical University, 20 Chazhong Road, Fuzhou, 350005 Fujian China; 6 Department of Geriatrics, National Regional Medical Center, Binhai Campus of the First Affiliated Hospital, Fujian Medical University, Fuzhou, China; 7 Branch of National Clinical Research Center for Aging and Medicine, Fujian Province, Fujian Provincial Clinical Research Center for Geriatric Hypertension Disease, Fuzhou, China

**Keywords:** Non-communicable diseases, Health belief, Behavior, Middle-aged, Older, Preventive health

## Abstract

**Background:**

The triadic relationship among the number of NCDs, preventive behaviors and health beliefs has not been fully explored, especially the role of health beliefs.

**Aims:**

To explore the association between the number of NCDs and preventive behaviors, as well as the mediating effect of health beliefs and its dimensions among middle-aged and older adults. Provide scientific evidence for developing targeted behavior intervention.

**Methods:**

Data from 2095 middle-aged and older adults who completed demographic information, health beliefs and preventive behaviors questionnaire. Mediation analysis was used to explore the association of health beliefs and its dimensions between the number of NCDs and preventive behaviors.

**Results:**

Health beliefs and self-efficacy positively impacted preventive behaviors, whereas perceived severity, while perceived barriers had negative effects. Health beliefs (*β* = − 0.1809, 95% CI − 0.2658 to − 0.0960) and its dimensions(Perceived barriers:*β* = − 0.0881, 95% CI − 0.1533 to − 0.0232, self-efficacy: *β* = − 0.2706, 95% CI − 0.3592 to − 0.1892) partially mediated the associations between the number of NCDs and preventive behaviors. The negative mediation effects indicates that as the number of NCDs increases, preventive behaviors decrease, partly due to a decline in health beliefs and self-efficacy, as well as an increase in perceived barriers (scored inversely, meaning higher barriers). These mediation pathways exhibited modest strength, highlighting the importance of health beliefs on behavior change.

**Conclusions:**

An increasing number of NCDs is associated with reduced engagement in preventive behaviors. Health beliefs and its dimensions play a partial mediating role in this relationship. Effective intervention targeting health beliefs may help promote positive behavioral changes.

**Supplementary Information:**

The online version contains supplementary material available at 10.1007/s40520-025-02939-3.

## Introduction

The aging rate in China is accelerating, and the prevalence of chronic non-communicable diseases (NCDs) has become a major public health issue [1]. Although people’s life expectancy has been extended, their overall health has declined [2]. The Sixth National Health Service Survey indicates that major chronic diseases, such as cardiovascular diseases, diabetes, and cancer account for over 90% of the economic burden of diseases in China [3]. With the growing prevalence of chronic diseases, the proportion of deaths attributable to these conditions has increased. In China, chronic diseases accounted for 88.5% of all deaths in 2019, a significant rise from the figure in 2015. This trend is echoed globally, as aging populations in various countries experience rising NCD prevalence and escalating healthcare costs [4–8].

Despite China launched nationwide campaigns promoting healthy lifestyles and policy interventions, substantial gaps persist in the effectiveness of prevention and management efforts [9]. Globally, similar challenges persist, as policies aimed at encouraging healthier lifestyles often produce inconsistent outcome [8, 10–12]. These measures underscore the urgent need for innovative, population-specific solutions to mitigate the NCD burden.

Engaging in preventive behaviors, defined as actions taken to maintain health and reduce disease risks [13], plays a pivotal role in managing NCDs and should considered as priority. Evidence proved that engaging in regular physical activity, maintaining a balanced diet, getting adequate sleep duration, maintaining a positive mood, and participating in routine health screenings are all crucial in combating NCDs. Researches consistently shows that individuals with multimorbidity face worse poor health outcomes compared with those without disease [14, 15]. This may be due to chronic diseases were often associated with more a rapid disability progression [16], functional limitation [17], and psychological distress [18]. Constrained by these conditions, patients often report lower levels of engagement in preventive behaviors and higher risk of depression [19, 20]. This indicates that the burden of NCDs may pose a significant barrier to individuals adopting a healthy lifestyle. Paradoxically, some studies suggest that a diagnosis of chronic disease can motivate individuals to adopt healthier behaviors [21, 22], while others report limited behavior change following such diagnoses [23, 24]. These conflicting findings highlight the complexity of understanding the factors that influence preventive behaviors, including demographic factors like gender, education, and income level [12], as well as psychological elements such as health risk perceptions [25] and health beliefs [26].

Health beliefs, conceptually defined as individuals’ perceptions regarding their vulnerability to illness and the benefits versus barriers of taking preventive actions, are a cornerstone of the Health Belief Model (HBM) [27]. It posits that an individual’s perceptions of susceptibility to disease, the severity of the disease, the benefits of taking action, and the barriers to action significantly influence their behaviors [28]. Research demonstrates that individuals with positive health beliefs are more likely to engage in preventive behaviors [26, 29–31]. In the context of NCDs, those who perceive higher susceptibility and acknowledge the benefits of prevention are more inclined to adopt healthy lifestyles.

In China, middle-aged and older adults are particularly vulnerable to the dual burden of physical and social challenges, contributing to a higher prevalence of NCDs and difficulties in sustaining preventive behaviors [32, 33]. This demographic is critical for intervention, as cumulative health risks manifest during this stage of life [34]. Research highlights that providing clear and accessible health education fosters confidence and encourages preventive behaviors [35, 36]. This suggests that tailored interventions addressing their unique health beliefs and barriers to preventive behaviors could significantly improve health outcomes.

Despite the established importance of health beliefs and preventive behaviors, the triadic relationship among the number of NCDs, preventive behaviors and health beliefs has not been fully explored. In particular, the mediating role of health beliefs warrants further investigation. Given the increasing burden of NCDs among middle-aged and older adults in China, understanding this interplay is essential. Such insights can inform the design of targeted health interventions and policies aimed at promoting healthier lifestyles and improving disease management in this vulnerable population. By elucidating how health beliefs mediate the relationship between the number of NCDs and preventive behaviors, this study can provide actionable strategies to enhance self-management capabilities and mitigate the public health burden of NCDs. To address this knowledge gap, this study investigates the association between the number of NCDs and preventive behaviors while examining the mediating effect of health beliefs among Chinese middle-aged and older adults. Specifically, we propose the following hypotheses:1. The number of NCDs negatively influences preventive behaviors; 2. Health beliefs mediate the relationship between the number of NCDs and preventive behaviors.

## Methods

### Study design

This study employed a cross-sectional survey conducted at a hospital in Fuzhou, Fujian, using convenience sampling from the Health Management Center. Prior to conducting the survey, researchers underwent training and an eligibility assessment designed by a nutritionist to ensure consistency and reliability. A standardized electronic questionnaire was used, supplemented by images of common local high-salt, high-fat foods, and vegetables, all validated by a nutritionist to ensure cultural and dietary relevance. These images were shown to all participants under standardized conditions to facilitate accurate responses. The study design has been detailed in our previously published works [37].

### Participants

Participants were initially selected if they met the following inclusion criteria: (1) aged ≥ 18 years; (2) proficiency in Chinese; (3) provision of informed consent and voluntary participation. However, only individuals aged 45 years or older were included in the final analysis, as this age group represents the primary target population for studying chronic disease burden. The exclusion criteria included: (1) unwillingness to participate; (2) unable to participate due to physical or mental disabilities or cognitive impairment.

A total of 4500 questionnaires were distributed; after excluding poorly completed responses (incomplete answers or response time under 8 min, based on pilot testing to ensure data quality), 4453 were recovered, resulting in a response rate of 98.9%. Among these, 2095 valid questionnaires were included in the final analysis, with 41.6% male and 58.4% female. Participants aged 45 ~ 59 years accounted for 81.0%. The majority of participants had attained university or above, constituting 69.0%. In terms of marital status, 92.2% of participants were married. The predominant working status was employed, encompassing 65.7%, and 22.0% of participants was retired. 8.0% of participants reported a monthly family income of less than 3000 RMB. Living in urban was reported by 76.8% of participants, and 19.4% in suburban, 3.8% in rural. Moreever, 98.8% and 95.2% of participants was hiving health insurance and living with others. The proportion of participants without NCDs was 44.0%, while 23.2% had one NCD, 16.8% had two NCDs, and 15.9% had three or more NCDs, detailed in Table S1.

## Measurements

### Demographic characteristics

The demographic characteristics examined included: sex, age(45–59, 60–74, ≥ 75), marital status(married, widowed/others), education level(elementary school, middle/high school, university or above), employment status(employed, unemployed, retired, others), monthly family income (RMB) categorized as follows(≤ 3000 yuan, 3001–6000 yuan, 6001–9,999 yuan, and ≥ 10,000 yuan), health insurance coverage(yes/no), region of residence(rural/suburban/urban), living arrangement (living alone or with the family/colleagues/others).

For the assessment of NCDs, we provided a list of common conditions categorized into the following groups: (1) cardiovascular diseases (such as heart attacks and strokes); (2) cancers; (3) chronic respiratory diseases (such as chronic obstructive pulmonary disease and asthma); (4) diabetes; (5) hyperlipidaemia; and (6) musculoskeletal disorders (such as rheumatoid arthritis, intervertebral disc disease). Additionally, “other” option was provided to allow participants to specify any NCDs not explicitly listed. Participants were instructed to indicate whether they had received a formal diagnosis from healthcare professionals for each of these NCDs. Subsequently, a cumulative count of the reported NCDs was calculated for each participant. Multimorbidity was defined as the concurrent presence of two or more NCDs within a single individual. Based on the number of self-reported NCDs by participants, four distinct classifications were established, namely 0, 1, 2, ≥ 3.

### Health belief scale

Health belief scale were assessed across six dimensions based on the Health Belief Model, included 26 items: perceived susceptibility (3 items), perceived severity (5 items), perceived benefits (4 items), perceived barriers (7 items), cues to action (4 items), and self-efficacy (3 items). These dimensions were categorized to capture the comprehensive psychological determinants of preventive behaviors, as outlined by the HBM framework. Each dimension addresses a specific aspect of decision-making in health behavior: perceived susceptibility reflects individuals’ beliefs about their risk of developing or worsening NCDs; perceived severity measures the perceived seriousness of the consequences of NCDs; perceived benefits evaluate the perceived advantages of engaging in preventive behaviors, while perceived barriers identify obstacles to adopting such behaviors; cues to action assess external or internal triggers that motivate health-related actions; self-efficacy measures confidence in one’s ability to perform preventive behaviors effectively.

Responses were scored on a 5-point Likert scale, ranging from 1 (strongly disagree) to 5 (strongly agree), except for perceived barriers which were reverse-coded. The score ranged 26 ~ 130, with higher scores indicating stronger health beliefs.

### Preventive behaviors scare

The study assessed sixteen preventive behaviors, which can be primarily divided into two categories: (1) Healthy behaviors: health checkup, fruit and vegetable intake, physical activity; (2) Unhealthy behaviors: high-salt diet (salt ≥ 5 g/day), high-fat diet or fried, baked food intake, skipping breakfast, overeating, sedentary living or working, smoking, heavy alcohol-taking, staying up late, depression, losing one’s temper, stress, delayed medical treatment when getting sick, non-compliance with instruction of doctors. Mental health behaviors, such as managing depression and stress, are essential components of chronic disease prevention owing to their modifiable nature and significant impact on disease progression. Incorporating these behaviors into preventive strategies reflects a holistic approach that aligns with contemporary frameworks emphasizing the integration of physical and mental health. A 4-point Likert scale was used to measure the frequency of these behaviors, including always, occasionally, seldom, never. The scale ranging from 1 (always) to 4 (never) for unhealthy behaviors, and reverse order for healthy behaviors. The overall score was calculated by summing the scores of all items. A higher score indicates healthier behaviors.

### Statistical analyses

AMOS 25.0 and SPSS 25.0 software was used for statistical analysis in this study, *P* < 0.05 was defined as a statistically significant difference. The steps are as follows: (1) Cronbach’s α coefficient was utilized to assess the reliability of the instrument. Exploratory and confirmatory factor analyses were conducted to evaluate the validity. The assessment of both reliability and validity adhered to the criteria outlined in this study [38]. (2) Correlation analysis includes the key variables in the mediation model and preliminarily explores their relationships. (3) Multiple linear regression was used to analyze the association between the number of NCDs and preventive behaviors. We assessed multicollinearity using the variance inflation factor (VIF). All variables exhibited VIF values below 5, indicating the absence of significant multicollinearity. (4) Referring to the hypothesized mediation model and test method [39, 40], the PROCESS and Bootstrap were performed to examine the mediating effect of health beliefs(model 1) and its dimensions(model 2) between the number of NCDs and preventive behaviors (repeated sampling was set 5000 times, and the confidence interval was set at 95%). In the mediation model 1, variables that were found to be significant in the multiple linear regression analysis (gender, age, employment status, education level) were included as covariates for adjustment. In the mediation model 2, gender, age, education levels were included as covariates for adjustment. First, the path from the number of NCDs to preventive behaviors (path c) was significant; third, controlling for number of NCDs, the path from health beliefs and its dimensions to preventive behaviors (path b) was also significant; and finally, the direct effect of health beliefs and its dimensions(a*b) in the association between the number of NCDs and preventive behaviors was significant (the 95% CI did not include 0).

## Results

### Reliability and validity analysis

The Cronbach’s α coefficient of the HB was 0.793, and the six dimensions of the HB yielded a Cronbach’s α coefficient of 0.650, 0.839, 0.905, 0.775, 0.779 and 0.848. The Cronbach’s α coefficient of the preventive behaviors was 0.752. These results indicated the reliability of the subscale was acceptable. The Kaiser–Meyer–Olkin (KMO) measure of sampling adequacy was 0.893, indicating a high level of suitability for factor analysis. Bartlett’s test of sphericity was also significant (*χ*^2^ = 23,406.757, df = 325, *P* < 0.001), supporting the appropriateness of the data for factor analysis.

HBM constructs were correlated with each other, and based on the relationships, an empirical model was built, as depicted in Figure S1. The results of confirmatory factor analysis indicate that the goodness of fit indices was as following: *χ*^2^/df = 4.053, Root Mean Square Error of Approximation (RMSEA) = 0.038, Standardized Root Mean square Residual (SRMR) = 0.034, Goodness of Fit Index (GFI) = 0.962, Normed Fit Index (NFI) = 0.955, Comparative Fit Index (CFI) = 0.965, Tucker Lewis index (TLI) = 0.957. Although the *χ*^2^/df was slightly above 3, all other fit indices perform very well, indicating overall structural validity was good, and the chosen items may represent HBM constructs. And the Average Variance Extracted (AVE) values fall within the range of 0.334 to 0.685, and the Composite Reliability (CR) values lie between 0.660 and 0.897. Although the values of AVE and CR are slightly lower than the conventional standards (0.5 and 0.7 respectively), the other model fitting indicators and the results of reliability analysis are satisfactory, indicating that the overall adaptability of the model is relatively high. These analyses were detailed in Table S2.

### Correlations among number of NCDs, health beliefs, and preventive behaviors

Among 2095 participants, the average score of preventive behaviors was 43.86 ± 5.98 and the average score of health beliefs was 99.29 ± 8.90. The Spearman’s *r* correlation analysis indicated that the number of NCDs had negative correlation with preventive behaviors(*r* = − 0.072, *P* < 0.01) and health beliefs(*r* = − 0.110, *P* < 0.01). Moreover, health beliefs showed positive correlation with preventive behaviors (*r* = 0.336, *P* < 0.01) (Table 1). Further Pearson’s *r* correlation analysis revealed that preventive behaviors were positively correlated with most dimensions of health beliefs, including perceived benefits (*r* = 0.137, *P* < 0.01), perceived barriers (*r* = 0.376, *P* < 0.01), cues to action (*r* = 0.215, *P* < 0.01), and self-efficacy (*r* = 0.419, *P* < 0.01), but negatively correlated with perceived severity (*r* = − 0.090, *P* < 0.01). Additionally, health beliefs were significantly and positively correlated with all its dimensions (*P* < 0.01). Significant interrelationships were also observed among these subdimensions, with the strongest correlations evident between perceived susceptibility and perceived severity (*r* = 0.479, *P* < 0.01), perceived susceptibility and perceived benefits (*r* = 0.465, *P* < 0.01), and cues to action and self-efficacy (*r* = 0.535, *P* < 0.01).

**Table 1:** Correlation analysis between number of NCDs, preventive behaviors and health beliefs

Variables	M(SD)	1	2	3	4	5	6	7	8	9
1.Number of NCDs	/	1.000								
2.Preventive behaviors	43.86 ± 5.98	− 0.072^**^	1.000							
3.Health beliefs	99.29 ± 8.90	− 0.110^**^	0.336^**^	1.000						
4.Perceived susceptibility	12.43 ± 1.77	− 0.087^**^	0.041	0.543^**^	1.000					
5.Perceived severity	20.04 ± 3.33	− 0.050^*^	− 0.090^**^	0.517^**^	0.479^**^	1.000				
6.Perceived benefits	18.17 ± 1.96	− 0.118^**^	0.137^**^	0.670^**^	0.465^**^	0.399^**^	1.000			
7.Perceived barriers	20.51 ± 5.11	− 0.049^*^	0.376^**^	0.467^**^	− 0.081^**^	− 0.254^**^	0.036	1.000		
8.Cues to action	16.35 ± 2.03	− 0.015	0.215^**^	0.601^**^	0.327^**^	0.328^**^	0.442^**^	0.021	1.000	
9.Self-efficacy	11.80 ± 1.91	− 0.117^**^	0.419^**^	0.617^**^	0.223^**^	0.228^**^	0.380^**^	0.172^**^	0.535^**^	1.000

*P < 0.05, **P < 0.01, ***P < 0.001

Perceived barriers are scored inversely, with a higher score indicating fewer perceived barriers and a lower score indicating more perceived barriers

### Multiple linear regression analysis of factors influencing preventive behaviors

As shown in Table 2, Model 1 indicates a significant negative association between having 2 NCDs (*β* = − 0.050, *P* = 0.032) or ≥ 3 NCDs (*β* = − 0.081, *P* = 0.001) and preventive behaviors. However, after adjusting for health beliefs in Model 2 and their six dimensions in Model 3, the associations between the number of NCDs and preventive behaviors were no longer statistically significant(*P *> 0.05), suggesting that health beliefs and its dimensions may mediate this relationship. The inclusion of health beliefs in Model 2 increases the adjusted *R*^2^ from 0.006 in Model 1 to 0.130, and further to 0.286 in Model 3. This substantial improvement in explanatory power indicates that health beliefs have a significantly stronger influence on preventive behaviors compared to merely considering the number of NCDs. Moreover, the incremental contribution of the six dimensions of health beliefs to the model fit is greater than the overall effect of health beliefs alone.

**Table 2:** Multiple linear regression analysis of preventive behaviors (N = 2095)

Variables	Model 1	Model 2	Model 3	Model 4	Model 5
	*β*	*β*	*β*	*β*	*β*
Number of NCDs (0 as reference)					
1	− 0.008	0.024	0.027	− 0.005	0.007
2	− 0.050*	− 0.027	− 0.022	− 0.063**	− 0.047*
≥ 3	− 0.081**	− 0.040	− 0.020	− 0.107***	− 0.067***
Health beliefs		0.356***	/	0.358***	/
Perceived susceptibility			0.028		0.027
Perceived severity			− 0.129***		− 0.088***
Perceived benefits			0.002		0.010
Perceived barriers			0.284***		0.268***
Cues to action			0.052*		0.050*
Self-efficacy			0.366***		0.340***
Constants	44.229***	20.540***	24.718***	12.824***	17.486***
Adjusted *R*^2^	0.006	0.130	0.286	0.220	0.331
*F*	4.955**	79.436***	94.321***	30.529***	42.408***

*P < 0.05, **P < 0.01, ***P < 0.001

Perceived barriers are scored inversely, with a higher score indicating fewer perceived barriers and a lower score indicating more perceived barriers. Model 1 was an unadjusted model. Model 2 and 3 were adjusted for health beliefs and six dimensions based on the HBM (perceived susceptibility, perceived severity, perceived benefits, perceived barriers, cues to action, self-efficacy). Model 4 and 5 were further adjusted for demographic characteristics (sex, age, education, marital status, employment status, monthly family income, region of residence, health insurance coverage, living arrangement)

In Model 4, further adjustment for demographic characteristics revealed that having 2 NCDs (*β* = –0.063, *P* = 0.003) or ≥ 3 NCDs (*β* = − 0.107, *P* < 0.001) remained significantly associated with preventive behaviors. In Model 5, which adjusts for both health beliefs and demographics, these associations remained significant, with an adjusted *R*^2^ of 0.331. However, the incremental improvement from demographic adjustments was relatively modest compared to the substantial impact of health beliefs observed in Model 3, indicating that demographic factors play a supplementary role. The results detailed in Table S3.

These findings underscore the dominant role of health beliefs and their dimensions in shaping preventive behaviors, while demographic factors provide additional context.

### Mediation test for health beliefs

We constructed two mediation models to evaluate the role of health beliefs in the relationship between the number of NCDs and preventive behaviors (Table 3). In model 1, the result showed that Health beliefs were found to partially mediate this relationship, accounting for 23.39% of the total effect. In other words, 23.39% of the effect of multiple NCDs on preventive behaviors is mediated through health beliefs, whereas the remaining portion is attributable to factors outside the mediation pathway.

**Table 3:** Mediation analysis of the relationship between the number of NCDs and preventive behaviors

Effect	Path	*Coef(β)*	*S.E*	*t*	*P*	BootLLCI	BootULCI
Model 1							
Total effect, c		− 0.7734	0.1153	− 6.7085	< 0.001	− 0.9995	− 0.5473
Direct effect, c’		− 0.5924	0.1072	− 5.5260	< 0.001	− 0.8027	− 0.3822
Indirect effect, a*b		− 0.1809	0.0433			− 0.2658	− 0.0960
Ratio of indirect to total effect mediated (a*b/c)						23.39%	
Model 2							
Total effect, c		− 0.7342	0.1140	− 6.4385	< 0.001	− 0.9578	− 0.5106
Direct effect, c’		− 0.3609	0.0998	− 3.6148	0.0003	− 0.5566	− 0.1651
Indirect effect, a*b	Total	− 0.3733	0.0612			− 0.4927	− 0.2551
	Path 1: NCD → PSU → PB	− 0.0097	0.0082			− 0.0284	0.0037
	Path 2: NCD → PSE → PB	0.0028	0.0112			− 0.0191	0.0265
	Path 3: NCD → PBE → PB	− 0.0024	0.0110			− 0.0240	0.0200
	Path 4: NCD → PBA → PB	− 0.0881	0.0332			− 0.1533	− 0.0232
	Path 5: NCD → CUE → PB	− 0.0053	0.0070			− 0.0217	0.0065
	Path 6: NCD → SE → PB	− 0.2706	0.0432			− 0.3592	− 0.1892
Ratio of indirect to total effect mediated (a*b/c)						50.84%	

Model 1: examining the mediating effect of health beliefs, and adjusted for sex, age, education and employment status; Model 2: examining the mediating effects of dimensions of health beliefs, adjusted for sex, age, education.

*NCD* non-communicable disease, *PSU* perceived susceptibility, *PSE* perceived severity, *PBE* perceived benefits, *PBA*, perceived barriers, *CUE* cues to action, *SE* self-efficacy, *PB* preventive behaviors

Perceived barriers are scored inversely, with a higher score indicating fewer perceived barriers and a lower score indicating more perceived barriers

**Fig. 1:**
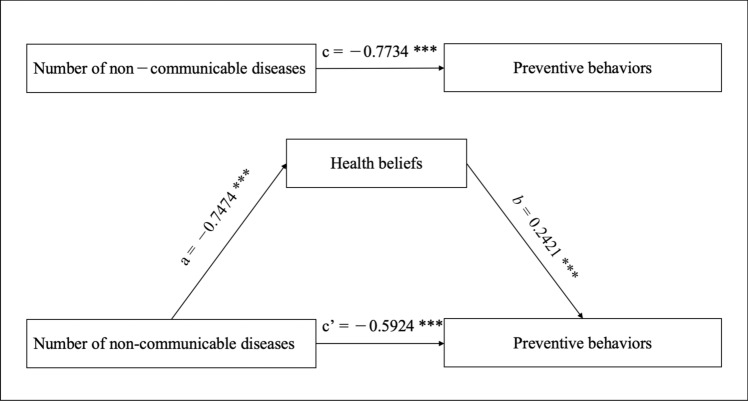
The mediation model for the number of NCDs, preventive behaviors, and health beliefs, *P < 0.05, **P < 0.01, ***P < 0.001

**Fig. 2:**
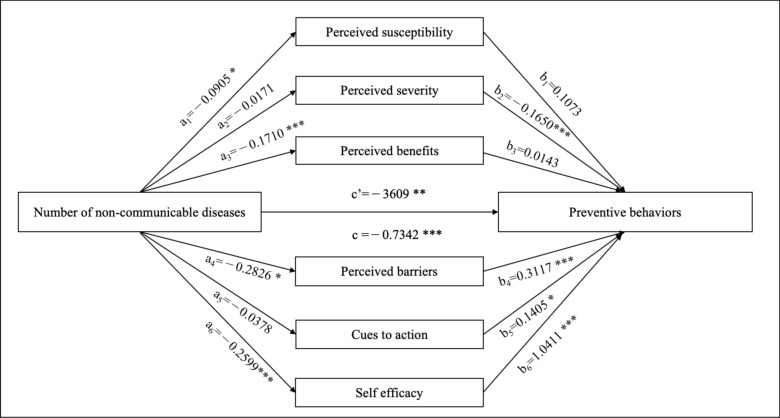
The parallel mediation model for the number of NCDs, preventive behaviors, and dimensions of health beliefs, *P < 0.05, **P < 0.01, ***P < 0.001. Perceived barriers are scored inversely, with a higher score indicating fewer perceived barriers and a lower score indicating more perceived barriers

To further explore the individual contribution of each dimension, we also conducted a parallel mediation analysis to examine the unique contributions of each dimension of health beliefs (perceived susceptibility, perceived severity, perceived benefits, perceived barriers, cues to action, and self-efficacy) to preventive behaviors. In model 2, there were two paths were significant (the 95% *CI* did not include 0). Path 4 was “NCD → PBA → PB”, which indicated that there was a mediating effect of PBA between NCD and PB with a mediation effect of − 0.0881(*SE* = 0.0332, 95% CI − 0.1533 to − 0.0232). Path 6 was “NCD → SE → PB” which indicated that there was a mediating effect of SE between NCD and PB with a mediation effect of − 0.2706(*SE* = 0.0432, 95% CI − 0.3592 to − 0.1892). The negative effect value means that there existed a suppression effect in two paths. Results indicated that the number of NCDs influences preventive behaviors, with health beliefs and its six dimensions mediating part of this effect. However, even after controlling for health beliefs and its six dimensions, the number of NCDs continues to have a direct influence on preventive behaviors (Figs. 1 and 2).

## Discussion

The study examined the relationship between number of NCDs and preventive behaviors among middle-aged and older adults in southern China, and explored the mediating effect of health beliefs and its dimensions in this relationship. By identify health beliefs as a mediating factor, the study sheds light on potential mechanisms and intervention targets. Our results showed that health beliefs played a mediating role in the associations between number of NCDs and preventive behaviors. Furthermore, the results showed that there were multiple mediating relationships among health beliefs, the number of NCDs, and preventive behaviors. The number of NCDs was not only directly related to preventive behaviors, but also negatively correlated to preventive behaviors through the mediating effect of PBA and SE (path 4, path 6 in Model 2, Table 4).

This study identified significant correlations between the number of NCDs and preventive behaviors. A study conducted in northeastern China found that consumption of fruits associated with risks of multimorbidity, eating rarely had a higher risk of multimorbidity [41]. Another study showed that older adults with two or more chronic diseases engage in less physical activity than those without disease [14]. It is a truism that NCDs were related to the negative effect on the body and spirit, poor prognosis with complications and disabilities. With the increase of the number of chronic diseases, patients had significantly increased functional disability [42], higher risk of depression [43], greater likelihood of possible sarcopenia [44], poorer quality of life [3].

Interestingly, we found that a single chronic disease had no significant effect on preventive behaviors. This finding aligns with the mixed evidence in existing literature regarding behavior changes following the diagnosis of a chronic disease. For example, a previous study demonstrated that a diagnosis of diabetes that negatively affects physical activity [23]. Similarly, a large majority of middle-aged and older smokers continued to smoke after diagnosis with a major chronic disease [24]. However, other studies suggest that opposite, with certain chronic diseases promoting positive health behavior changes. For instance, prior research has shown that Chinese middle-aged and elderly people diagnosed with hypertension tend to reduce smoking and alcohol consumption, increasing exercise frequency, and utilize health services more actively [45]. Another study involving 40,490 participants revealed that those with cardiovascular diseases were more likely to adopt healthy lifestyle behaviors [46]. Furthermore, individuals who have experienced cardiovascular disease may improve their lifestyle habits to a greater degree than those without disease [47]. The discrepancy in findings may be attributed to various factors, such as differences in the types of chronic disease, patient perceptions, and contextual influences. For example, inaccurate self-assessment among patients with chronic diseases [48] and negative emotional responses such as denial or fatalism [49], both of which impede behavior change. These findings suggest the need to delve deeper into the social and cultural contexts that shape individuals’ responses to chronic illness. Former study showed social support plays a crucial role in influencing behavior change, whereas those lacking such support may not perceive the necessity for change [23]. Moreover, cultural beliefs and norms significantly shape perceptions of health and illness. In some cultures, chronic diseases are viewed as an inevitable part of aging, fostering fatalistic attitudes that discourage proactive health measures [50]. Furthermore, political beliefs and religious beliefs have been verified as direct and indirect predictor of preventive behaviors, such as COVID-19 vaccination uptake [51]. Chronic diseases often induce stress, and the coping strategies individuals employ can affect their engagement in preventive behaviors. Maladaptive coping strategies may hinder the adoption of health-promoting actions [52, 53]. These findings offer valuable insights for designing preventive behavior interventions targeting at individuals with chronic diseases. Interventions could focus on leveraging psychosocial resources, such family and community support, to help individuals effectively cope with their new health challenges. Additionally, integrating culturally sensitive approaches and addressing emotional and psychological barriers may further enhance the effectiveness of such interventions.

We focused on exploring the mediating role of health beliefs in the number of chronic diseases and preventive behaviors, and found that individuals affected by NCDs commonly report diminished health beliefs. Related researches founds that lack of knowledge around behavior and poor health literacy has been concerned as a key barrier to behavioral change [54, 55]. The HBM emphasizes that the first stages in lowering lifestyle risk are identifying the health issue and being aware of its possible effects [56]. Numerous empirical studies have demonstrated that the promotion of health beliefs plays a pivotal role in encouraging preventive behaviors. A research about middle-aged and elderly people in Chongqing found that health beliefs can explain up to 39.30% of lifestyle variations [57]. One study conducted in Korean older adults showed that health beliefs directly affect health promotion behavior, and affect successful aging through the mediation of health promotion behavior [29]. Another study also found that health belief model is a strong predictor of the likelihood of eating nutrient-rich foods in American adults [58]. Our results showed that health beliefs played a mediating role in the association between the number of NCDs and preventive behaviors, accounting for 26.39% of the total effect. This percentage indicates a moderate mediating effect, suggesting that health beliefs serve as an important pathway through which the number of NCDs impacts preventive behaviors. Compared to similar studies, where the mediation effects of psychosocial factors on health behaviors ranged between 14.00% and 28.57% [59–61], the current findings highlight the significant role of health beliefs in shaping preventive behaviors in this population.

The study further distinguished the mechanisms underlying the relationship between the number of chronic diseases and preventive behaviors and found that perceived barriers and self-efficacy plays prominent roles(*P* < 0.05). The findings suggest a complex interplay between these factors, which may be explained below: First, the study revealed that a significant negative relationship between the number of NCDs and perceived barriers, indicating that as the number of NCDs increase, the score for perceived barriers decreases. Given that perceived barriers are inverse-scored, with a higher score indicating fewer perceived barriers and a lower score indicating more perceived barriers. Additionally, perceived barriers were positively associated with preventive behaviors, implying that individuals who perceive fewer actual barriers are more likely to engage in preventive behaviors. These findings underscore the critical role of perceived barriers in shaping preventive behaviors among individuals with chronic conditions. Individuals who with worsened conditions not only generate heightened physical distress but also necessitate intricate therapeutic interventions, engenders challenges such as detrimental polypharmacy and overwhelming medical expense burdens, which contribute to an increased perception of barriers [62–64]. Consequently, these barriers negatively impact their ability to consistently engage in preventive behaviors. This finding aligns with the HBM, which posits that lower perceived barriers increase the likelihood of health behavior adoption [65]. To support individuals with chronic diseases in overcoming these barriers, tailored interventions should address both practical and psychological aspects. Practically, simplifying healthcare recommendations, reducing financial burdens, and improving access to preventive services can help mitigate actual barriers. Psychologically, providing targeted health education to enhance patients’ understanding of preventive behaviors and their benefits may further reduce perceived barriers. Peer support programs and community-based interventions have also been shown to assist patients in navigating health-related challenges, thereby improving their engagement in preventive behaviors.

Second, self-efficacy emerged as a significant positive mediator in the relationship between the number of NCDs and preventive behaviors. The results indicate that as the number of chronic diseases increases, individuals may develop greater confidence in their ability to perform preventive behaviors, likely due to accumulated experience with disease management or exposure to health education. Enhanced self-efficacy, in turn, promotes engagement in preventive behaviors, consistent with previous research. Studies have demonstrated that self-efficacy is a critical determinant of health behavior adoption, influencing behaviors such as medication adherence, weight management, and osteoporosis preventive behaviors [66–68]. This finding underscores the importance of designing interventions that actively foster self-efficacy by providing patients with opportunities for small, achievable successes, along with positive reinforcement and peer support.

These findings collectively demonstrate that individuals’ health beliefs significantly influence their choices to lead healthy lives. Our study highlights the critical role of health beliefs in shaping preventive behaviors among middle-aged and older adults, underscoring the necessity for targeted interventions aimed at enhancing self-efficacy and mitigating perceived barriers. To enhance the real-world applicability of these findings, integrating self-efficacy enhancement into policy and public health initiatives is essential. For instance, healthcare policies could support the integration of self-efficacy training into routine care through government-funded programs. Community-based public health campaigns could focus on building confidence in adopting preventive behaviors by organizing skill-building workshops and personalized goal-setting sessions. Policymakers could also incentivize healthcare providers to deliver structured motivational support programs as part of primary care services. Collaborations between healthcare providers and community organizations can foster the development and delivery of HBM-based programs, such as peer support groups and group exercise initiatives, empowering individuals to better manage their health. Simplifying behavioral recommendations and addressing structural challenges, including limited healthcare access and inadequate social support, can further facilitate preventive actions. Health education programs should prioritize fostering confidence and competence in adopting preventive behaviors, integrating self-efficacy strategies into public health campaigns. For example, using relatable success stories and role models to illustrate achievable health goals can inspire behavioral change. Moreover, public health policies can improve access to resources like affordable, nutritious food and low-cost community fitness programs, creating environments that support healthier lifestyles. By addressing psychological barriers and emphasizing self-efficacy, these initiatives can more effectively promote sustainable preventive behaviors on a larger scale.

## Conclusion

There has been extensive research into the connections between NCDs, preventive behaviors, and health beliefs. However, not much attention has been given to the role of health beliefs in the relationship between the number of NCDs and preventive behaviors. This study found that among middle-aged and older individuals, health beliefs positively moderated the link between the number of NCDs and preventive behaviors, especially perceived barriers and self-efficacy. Stronger health beliefs can help mitigate the negative impact of NCDs and promote preventive behavior. In summary, higher health beliefs could be a valuable factor linking NCDs to better preventive behaviors. But the mechanism of health belief varies in different behavior types [69]. Health educators should pay attention to different relationship between different behaviors and health beliefs, and develop different strategies to encourage healthy behavior of the middle-aged and elderly.

## Limitations

This study has several limitations. First, the use of convenience sampling, with all data collected from a hospital-based research sample, limits the generalizability of the findings to the broader population. Participants in a hospital setting may differ from community-based or nationally representative samples in terms of health status, access to healthcare, and health beliefs, potentially influencing the results. Our sample may not fully represent individuals from lower socioeconomic backgrounds or those in rural areas. Future studies should consider employing random sampling methods or recruiting participants from diverse settings to enhance the representativeness of the findings. Second, as a cross-sectional survey, the significant correlation between variables does not indicate causality. Longitudinal studies or intervention-based designs are needed to confirm the temporal and causal relationships. Such as tracking a diverse cohort across different regions and socioeconomic backgrounds to examine how health beliefs and preventive behaviors evolve over time. Third, the health status was self-reported by participants, which introduces potential recall bias and subjectivity. Since our results are strongly associated with the severity of participants’ conditions, so it’s should be cautious about the comparison of study results with previous study that may use objective clinical measurements. Incorporating objective health data, such as medical records or biomarkers, in future research could provide more robust evidence.

## Supplementary Information

Below is the link to the electronic supplementary material.

Supplementary file1 (PDF 448 KB)

## Data Availability

The data of this study are available from the corresponding author on reasonable request.
