# Supplementary material for: Health beliefs mediates the association between the number of non-communicable diseases and preventive behaviors in middle-aged and older adults in southern China
Source: Aging Clin Exp Res. 2025 Feb 25;37(1):49. doi: 10.1007/s40520-025-02939-3 (PMC11850486; doi:10.1007/s40520-025-02939-3)
Supplement: Supplementary file 1 — Supplementary file1 (PDF 448 KB) [file 40520_2025_2939_MOESM1_ESM.pdf]

Article title: Health beliefs mediates the association between the number of non-communicable diseases and preventive behaviors in middle-aged and older adults in southern China

Journal name: Aging Clinical and Experimental Research

Author names: Yali Huang [1, 2], Tingjun Wang [1, 3, 4] , Huajun Wang [1], Yongjun Zeng [1], Liangdi Xie [1, 3, 4, 5, 6, 7]

Affiliation: [1] Fujian Hypertension Research Institute, The First Affiliated Hospital of Fujian Medical University, Fuzhou, 350005, China

[2] The School of Public Health, Fujian Medical University, Fuzhou, China

[3] Department of General Practice, National Regional Medical Center, Binhai Campus of the First Affiliated Hospital, Fujian Medical University, Fuzhou, China

[4] Department of General Practice, The First Affiliated Hospital of Fujian Medical University, Fuzhou, China

[5] Department of Geriatrics, The First Affiliated Hospital of Fujian Medical University, Fuzhou, China

[6] Department of Geriatrics, National Regional Medical Center, Binhai Campus of the First Affiliated Hospital, Fujian Medical University, Fuzhou, China

[7] Branch of National Clinical Research Center for Aging and Medicine, Fujian Province, Fujian Provincial Clinical Research Center for Geriatric Hypertension Disease, Fuzhou, China

Corresponding author: Liangdi, Xie; MD, PhD; Address: Department of Geriatrics, The First Affiliated Hospital of Fujian Medical University, 20 Chazhong Road, Fuzhou, Fujian 350005, China; Email: ldxield@163.com; Tel: 86-13509369323.

Table S1 Characteristics of study participants(N=2095).

Characteristics		Number (%)
Sex	Male	872(41.6)
	Female	1223(58.4)
Age(years)	45~59	1697(81.0)
	60~74	343(16.4)
	≥75	55(2.6)
Education levels	Elementary school	24(1.1)
	Middle/high school	626(29.9)
	University or above	1445(69.0)
Marital status	Married	1931(92.2)
	Widowed/others	164(7.8)
Working status	Employed	1376(65.7)
	Unemployed	67(3.2)
	Retired	461(22.0)
	Others	191(9.1)
Monthly family income (RMB)	＜3000	167(8.0)
	3000-6000	735(35.1)
	6000-10000	653(31.2)
	≥10000	540(25.8)
Region of residence	Rural	80(3.8)
	Suburban	406(19.4)
	Urban	1609(76.8)
Health insurance coverage	Yes	2070(98.8)
	No	25(1.2)
Living arrangement	Alone	101(4.8)
	With others	1994(95.2)
Number of non-communicable diseases	0	922(44.0)
	1	487(23.2)
	2	352(16.8)
	≥3	334(15.9)

Figure S1 Fitting model of health beliefs**.**

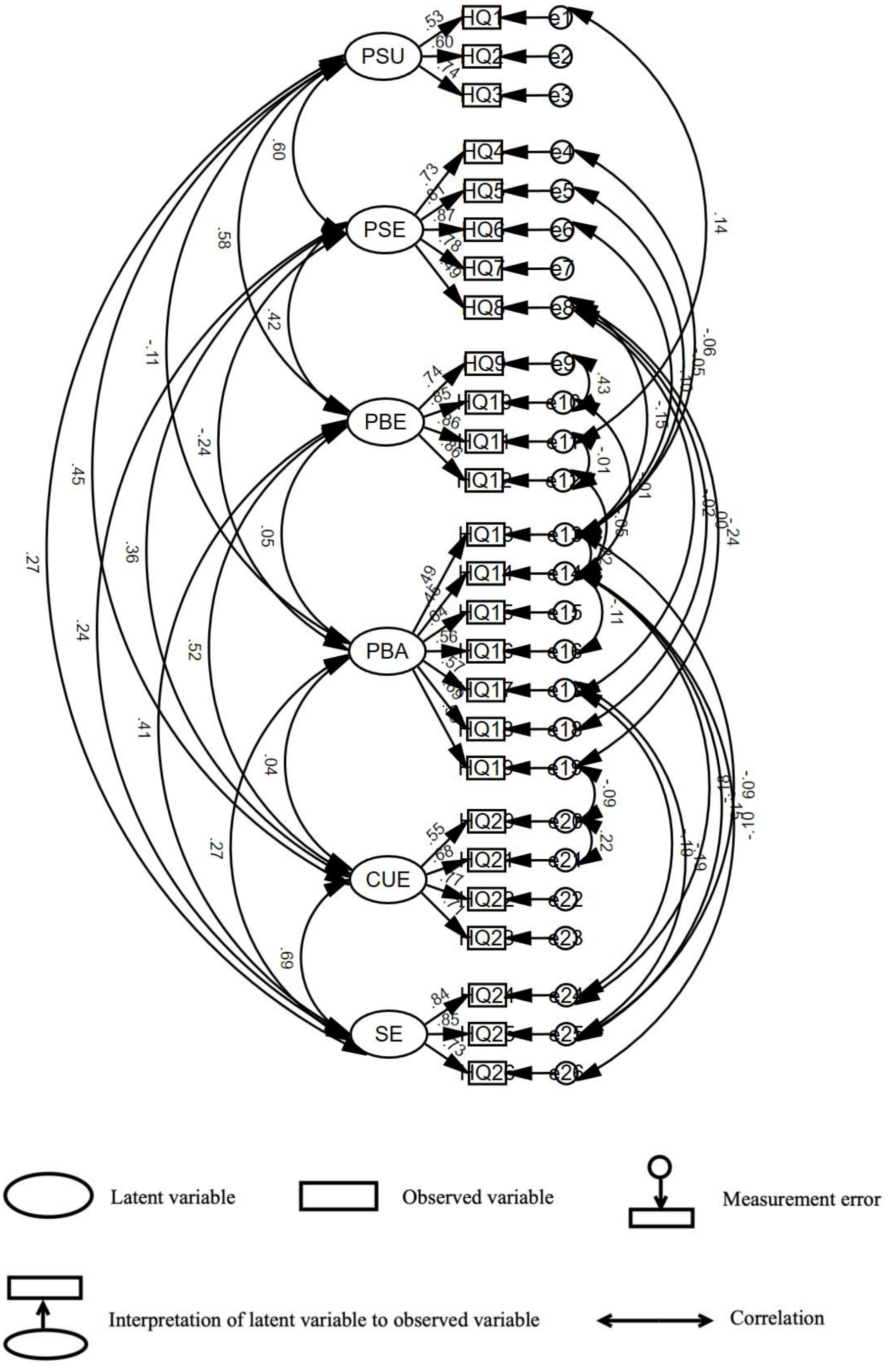

Note: An empirical model was built based on the correlation of health belief model constructs.

PSU: perceived susceptibility; PSE: perceived severity; PBE: perceived benefits; PBA: perceived barriers; CUE: cues to action; SE: self-efficacy.

HQ: health belief item.

Table S2 Reliability and validity analysis.

Items	Index	Criteria	Value
Internal consistency	Cronbach's α	>0.70	Health beleifs= 0.793; Perceived susceptibility = 0.650<br>Perceived severity=0.839; Perceived benefits=0.905<br>Perceived barriers=0.775; Cues to action=0.779<br>Self-efficacy=0.848; Preventive behaviors=0.752
Exploratory factor analyses	Kaiser-Meyer-Olkin (KMO)<br>Bartlett's test	KMO > 0.70<br>Bartlett's test< 0.05 ($P < 0.05$)	The results of the KMO and Bartlett's test were 0.893 and 23406.757, respectively. Additionally, the significance level of Bartlett's test was significiant ($\chi^2 = 23406.757$, df = 325, $P < 0.001$), indicating that performing EFA on this questionnaire is justified.
Confirmatory factor analyses: Fit indices	χ^2/df	<3	1062/262 = 4.053
	Root Mean Square Error of Approximation, RMSEA	<0.08	0.038
	Standardized Root Mean Square Residual, SRMR	<0.08	0.034
	Goodness of Fit Index, GFI	> 0.90	0.962
	Normed Fit Index, NFI	> 0.90	0.955
	Comparative Fit Index, CFI	>0.90	0.965
	Tucker Lewis index, TLI	>0.90	0.957
Convergent validity	Average Variances Extracted, AVE	>0.50	Perceived susceptibility=0.397; Perceived severity=0.558<br>Perceived benefits=0.685; Perceived barriers=0.334<br>Cues to action=0.478; Self-efficacy=0.655
	Composite Reliability, CR	>0.70	Perceived susceptibility=0.660; Perceived severity=0.860

	Perceived benefits=0.897; Perceived barriers=0.776
	Cues to action=0.783; Self-efficacy=0.850

Table S3 Multiple linear regression analysis of preventive behaviors.

Variables	Model 1		Model 2		Model 3		Model 4		Model 5	
	β	*P*	*β*	*P*	*β*	*P*	*β*	*P*	*β*	*P*
Number of NCDs (0 as Reference)										
1	− 0.008	0.734	0.024	0.286	0.027	0.183	− 0.005	0.795	0.007	0.735
2	− 0.050	0.032*	− 0.027	0.211	− 0.022	0.269	− 0.063	0.003**	− 0.047	0.015*
⩾3	− 0.081	0.001**	− 0.040	0.071	− 0.020	0.313	− 0.107	＜0.001**	− 0.067	＜0.001***
Health beliefs			0.356	＜0.001***	/		0.358	＜0.001***	/	
Perceived susceptibility					0.028	0.210			0.027	0.218
Perceived severity					− 0.129	＜0.001***			− 0.088	＜0.001***
Perceived benefits					0.002	0.923			0.010	0.655
Perceived barriers					0.284	＜0.001***			0.268	＜0.001***
Cues to action					0.052	0.030*			0.050	0.033*
Self-efficacy					0.366	＜0.001***			0.340	＜0.001***
Sex										
Male(Ref)										
Female							0.126	＜0.001***	0.105	＜0.001***
Age(years)										
45~59(Ref)										
60~74							0.188	＜0.001***	0.156	＜0.001***
≥75							0.132	＜0.001***	0.103	＜0.001***

Education levels				
Elementary school(Ref)				
Middle/high school	0.182	0.039*	0.200	0.015*
University or above	0.124	0.170	0.157	0.061
Marital status				
Married(Ref)				
Widowed/others	0.008	0.723	0.005	0.802
Working status				
Employed(Ref)				
Unemployed	0.002	0.930	− 0.001	0.955
Retired	0.073	0.007**	0.020	0.426
Others	0.011	0.577	0.001	0.973
Monthly family income (RMB)				
＜3000(Ref)				
3000-6000	− 0.025	0.528	− 0.012	0.740
6000-10000	− 0.003	0.933	0.003	0.929
≥10000	0.013	0.743	0.019	0.599
Region of residence				
Rural(Ref)				
Suburban	0.074	0.104	0.059	0.161
Urban	0.083	0.082	0.067	0.129

Health insurance coverage										
Yes(Ref)										
No							0.006	0.746	-0.003	0.864
Living arrangement										
Alone(Ref)										
With others							0.016	0.454	0.029	0.132
Constants	44.229	＜0.001***	20.540	＜0.001***	24.718	＜0.001***	12.824	＜0.001***	17.486	＜0.001***
Adjusted R^2	0.006		0.130		0.286		0.220		0.331	
F	4.955	0.002**	79.436***	＜0.001***	94.321	＜0.001***	30.529	＜0.001***	42.408	＜0.001***

* $P < 0.05$, ** $P < 0.01$, *** $P < 0.001$.

Note: Perceived barriers are scored inversely, with a higher score indicating fewer perceived barriers and a lower score indicating more perceived barriers.

Model 1 was an unadjusted model. Model 2 and 3 were adjusted for health beliefs and six dimensions based on the HBM (perceived susceptibility, perceived severity, perceived benefits, perceived barriers, cues to action, self-efficacy). Model 4 and 5 were further adjusted for demographic characteristics (sex, age, education, marital status, employment status, monthly family income, region of residence, health insurance coverage, living arrangement).
